# Non-thyroidal illness syndrome and the prognosis of heart failure: a systematic review and meta-analysis

**DOI:** 10.1530/EC-23-0048

**Published:** 2023-07-05

**Authors:** Xiaoyi Qi, Liangxian Qiu, Shijia Wang, Xiongbiao Chen, Qianwen Huang, Yixuan Zhao, Kunfu Ouyang, Yanjun Chen

**Affiliations:** 1 Departments of Cardiology, Peking University Shenzhen Hospital, Shenzhen, China; 2 Medical College, Shantou University, Shantou, China; 3 Department of Cardiovascular Surgery, Peking University Shenzhen Hospital, School of Chemical Biology and Biotechnology, State Key Laboratory of Chemical Oncogenomics, Peking University Shenzhen Graduate School, Shenzhen, China

**Keywords:** non-thyroidal illness syndrome, heart failure, prognosis, meta-analysis

## Abstract

**Background:**

Heart failure (HF) is a complex and multifactorial syndrome caused by impaired heart function. The high morbidity and mortality of HF cause a heavy burden of illness worldwide. Non-thyroidal illness syndrome (NTIS) refers to aberrant serum thyroid parameters in patients without past thyroid disease. Observational studies have indicated that NTIS is associated with a higher risk of all-cause mortality in HF. This meta-analysis aimed to investigate the association between NTIS and HF prognosis.

**Methods:**

Medline, Embase, Web of Science, and the Cochrane database were searched for any studies reporting an association between NTIS and HF prognosis from inception to 1 July 2022. A meta-analysis was then performed. The quality of studies was assessed using the Newcastle–Ottawa Scale. The heterogeneity of the results was assessed with *I*^2^ and Cochran's *Q* statistics. Sensitivity analysis and publication bias analysis were also conducted.

**Results:**

A total of 626 studies were retrieved, and 18 studies were finally included in the meta-analysis. The results showed that NTIS in HF patients was significantly associated with an increased risk of all-cause mortality and major cardiovascular events (MACE), but not with in-hospital mortality. The stability of the data was validated by the sensitivity analysis. There was no indication of a publication bias in the pooled results for all-cause mortality and MACE.

**Conclusions:**

This meta-analysis showed that NTIS was associated with a worse outcome in HF patients. However, the association between NTIS and in-hospital mortality of HF patients requires further investigation.

## Introduction

Heart failure (HF) is a complex, multifactorial clinical syndrome characterized by dyspnea and fluid retention caused by impaired systolic or diastolic cardiac function (1, 2). In 2017, there were 64.3 million cases of HF worldwide, an increase of 91.9% from 1990. Nearly half of the global increase occurred in China and India (1). According to the global burden of HF, the 1-year mortality risk is 15–30% and the 5-year mortality risk is as high as 75% in several populations (2). Additionally, the estimated annual global HF-related economic costs reached US $108 billion (1). Therefore, it is vital to identify new prognostic factors or treatment targets to reduce the HF-related burden of disease.

Non-thyroidal illness syndrome (NTIS) was first discovered in the 1970s and described as abnormal serum thyroid parameters in patients with acute illness but without prior thyroid disease. The most common abnormality is low triiodothyronine (T3) levels with normal thyroid-stimulating hormone levels (3). According to a previous observational study, NTIS may be present in up to 70% of critically ill patients and is associated with increased mortality (4). Furthermore, the results of a meta-analysis indicated that NTIS is most prevalent among HF patients. Pooled hazard ratios (HRs) from 41 studies showed that NTIS was independently associated with an elevated risk of cardiac and all-cause mortality in patients with cardiovascular disease (5). However, other researchers have found no correlation between NTIS and the HF prognosis (6, 7), and supporting evidence from large-scale prospective cohort studies is scarce.

Therefore, we performed this meta-analysis to explore the association between NTIS and the HF prognosis for various endpoints and subgroups. To our knowledge, this is the first meta-analysis to systematically review these associations.

## Methods

### Overall design

This meta-analysis was performed according to the Preferred Reporting Items for Systematic Review and Meta-Analyses (PRISMA) guideline 2020 (8). We registered this study at the international prospective register of systematic reviews website (Prospero CRD: 42022345038) (https://www.crd.york.ac.uk/PROSPERO).

### Search strategy and inclusion criteria

We conducted a comprehensive literature search of the Medline, Embase, Web of Science, and Cochrane databases to obtain studies focused on the association between NTIS and the HF prognosis, published from inception to 1 July 2022. The inclusion criteria were as follows: (i) HF patients were included in the study populations; (ii) cohort studies; (iii) studies investigating the association between NTIS and HF prognosis; (iv) study endpoints included all-cause mortality or major cardiovascular events (MACE), or in-hospital mortality; and (v) studies published in English. Our search strategy was therefore: (heart failure OR heart decompensation OR congestive heart failure OR HF) AND (non-thyroidal illness syndrome OR NTIS OR low T_3_ syndrome OR low triiodothyronine syndrome OR euthyroid sick syndrome OR ESS). Two reviewers (XYQ and YXZ) screened the titles and abstracts of the retrieved studies. The full texts of relevant studies were further evaluated, and reviews, case reports, animal experiments, and studies without prognosis information were excluded. A third reviewer (YJC) was consulted when there was a difference of opinion between the first two reviewers.

### Data extraction

The information extracted from the eligible studies included the author names, publication date, country, follow-up duration, sample size, study design, definition of NTIS, exclusion criteria, and endpoints. We also retrieved the effect size (odds ratio (OR), relative risk (RR), HR), with 95% confidence intervals (95% CIs) and adjusted covariates for multivariable analysis.

### Quality assessment

We used the Newcastle–Ottawa Scale (NOS) to evaluate the quality of the eligible cohort studies (9). The NOS is a nine-point scale that allocates points based on the selection of the cohort (0–4 points), comparability between exposure and control groups (0–2 points), and identification of outcome (0–3 points). Studies were categorized as high-, medium-, or low-quality if the NOS score was ≥7, 5–6, or ≤4 points, respectively. All the data extraction and quality assessment processes were checked by two independent reviewers (YXZ and XYQ).

### Statistical analyses

The RR, OR, or HR were extracted from the original studies. In studies that only gave survival curves, we calculated the crude HR using Engauge Digitizer 4.1 software (10, 11). Pooled ORs or HRs and 95% CIs were synthesized to estimate the association between NTIS and HF prognosis using a random-effect model according to the heterogeneity. The *I*² and Cochran’s *Q* statistics were used to assess heterogeneity. A *P*-value < 0.05 for *Q* statistic and *I*² > 50% was considered substantial heterogeneity. The sensitivity analysis was performed by omitting each study to evaluate the consistency of the pooled results. Publication bias was assessed using the funnel plot and Egger’s test. Obvious asymmetry of the funnel plot and a *P*-value < 0.05 for Egger’s test indicated significant publication bias. The trim and fill method would be used if publication bias existed. Subgroup analyses were conducted on disease onset of HF, NTIS definition, continent of origin of the study population, and source of HRs among different endpoints, that is, whether the HRs were reported or extracted from survival curves. A subgroup analysis according to MACE subtypes was also performed. All statistical analysis was performed using Stata software (Version 14.0). A two-sided *P*-value<0.05 was considered statistically significant.

## Results

### Characteristics of eligible studies

**Figure 1:**
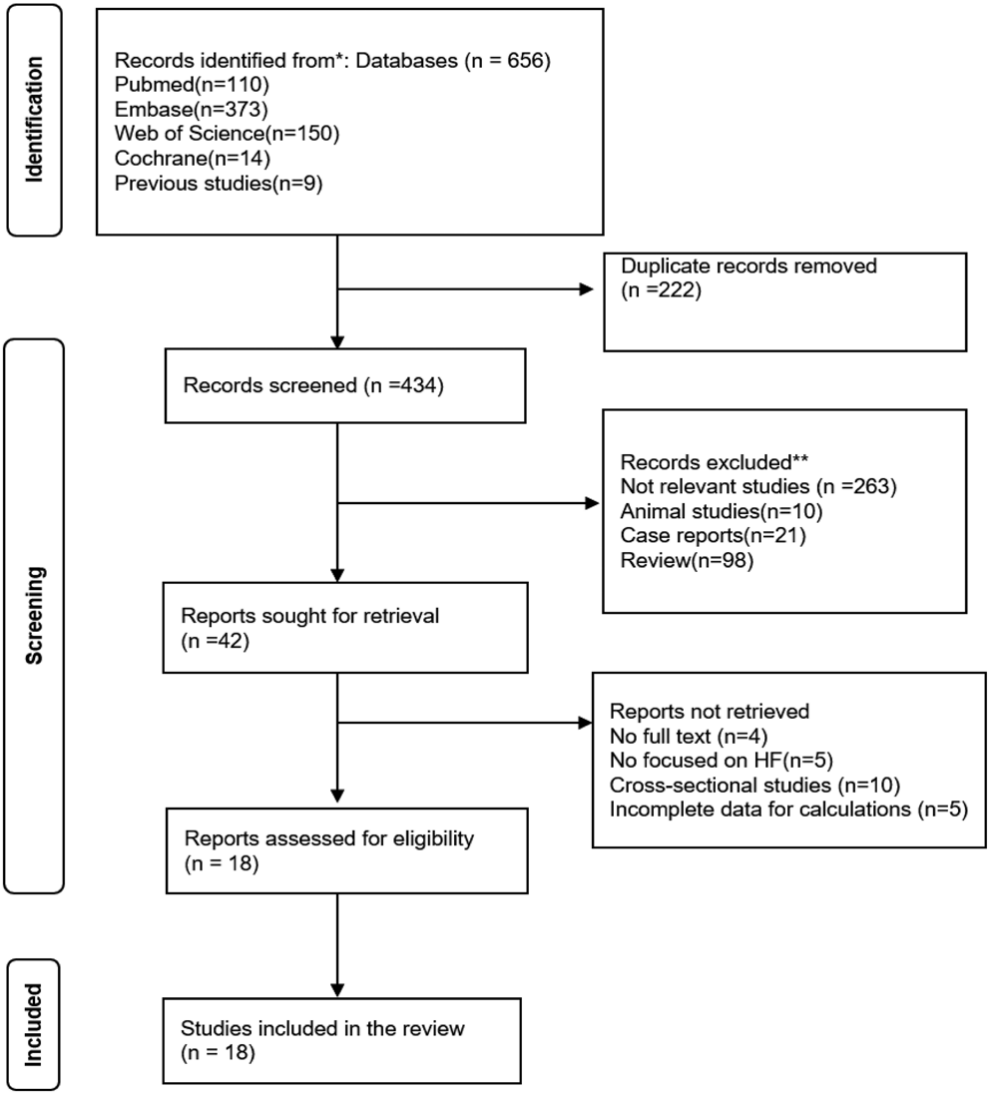
PRISMA flow chart for the selection of articles.

The initial database search identified 656 articles. After eliminating 222 duplicate studies, we screened the titles and abstracts of the remaining records. Studies not relevant to our topic (*n* = 263), animal studies (*n* = 10), case reports (*n* = 21), and reviews (*n* = 98), were excluded. Forty-two reports were retained for further examination. We excluded four further studies that were conference abstracts or for which we had no access to the full text, five studies that were not focused on HF patients, ten that were cross-sectional studies, and five that did not have data that would enable the determination of effect size. Finally, our meta-analysis included 18 cohort studies that examined the relationship between NTIS and HF prognosis. The workflow is shown in Fig. 1.

Details of the analyzed studies are presented in Table 1. The 18 eligible studies included 2 retrospective and 16 prospective cohort studies. The sample ranged from 59 to 956. Twelve studies focused on the association between NTIS and HF all-cause mortality. Eleven studies analyzed MACE in HF while only two studies focused on in-hospital mortality in HF. Five studies reported effect size as ORs and eight as HRs. We extracted HRs from the survival curves in the last five studies. Except for one study considered medium quality (23), other included studies were considered as high quality with scores > 7 points.

**Table 1:** Characteristics of included studies.

Study	Year	Country	Study type	Sample size	Endpoint	Effect estimates	NOS score
Opasich *et al*. (19)	1996	Italy	Prospective	199	MACE	OR	8
Rays *et al*. (20)	2003	Brazil	Prospective	69	All-cause mortality	OR	9
Passino *et al*. (21)	2009	Italy	Prospective	442	All-cause mortality/MACE	HR (SC)	8
Kozdag *et al*. (22)	2010	America	Prospective	334	MACE	HR (SC)	8
Cikrikcioglu *et al*. (23)	2012	Turkey	Prospective	176	MACE	OR	6
Frey *et al*. (24)	2013	Germany	Prospective	641	All-cause mortality	HR	7
Chuang *et al* (25)	2014	China	Prospective	106	All-cause mortality	HR	8
Chen *et al* (26)	2015	China	Retrospective	224	MACE	HR (SC)	8
Okayama *et al* (27)	2015	Japan	Retrospective	270	All-cause mortality/MACE/in-hospital mortality	OR	8
Hayashi *et al* (7)	2016	Japan	Prospective	274	MACE	HR	8
Terlizzese *et al*. (28)	2017	Italy	Prospective	712	All-cause mortality/MACE	HR	8
Kannan *et al* (29)	2018	America	Prospective	1365	All-cause mortality	HR	8
Sato *et al*. (30)	2018	Japan	Prospective	911	All-cause mortality	HR	8
Fraczek-Jucha *et al.* (31)	2019	Poland	Prospective	59	MACE	HR (SC)	7
Secco *et al*. (32)	2020	France	Prospective	353	All-cause mortality/in-hospital mortality	HR (SC)	8
Asai *et al*. (33)	2020	Japan	Prospective	956	All-cause mortality/MACE	OR	8
Iacoviello *et al*. (34)	2020	Italy	Prospective	762	All-cause mortality/MACE	HR	8
Zhao *et al.* (35)	2021	China	Prospective	594	All-cause mortality/in-hospital mortality	HR	8

HR, hazard ratio; HR (SC), hazard ratio from survival curves; NOS, Newcastle–Ottawa scale; MACE, major cardiovascular events; OR, odds ratio.

### Association between NTIS and all-cause mortality

**Figure 2:**
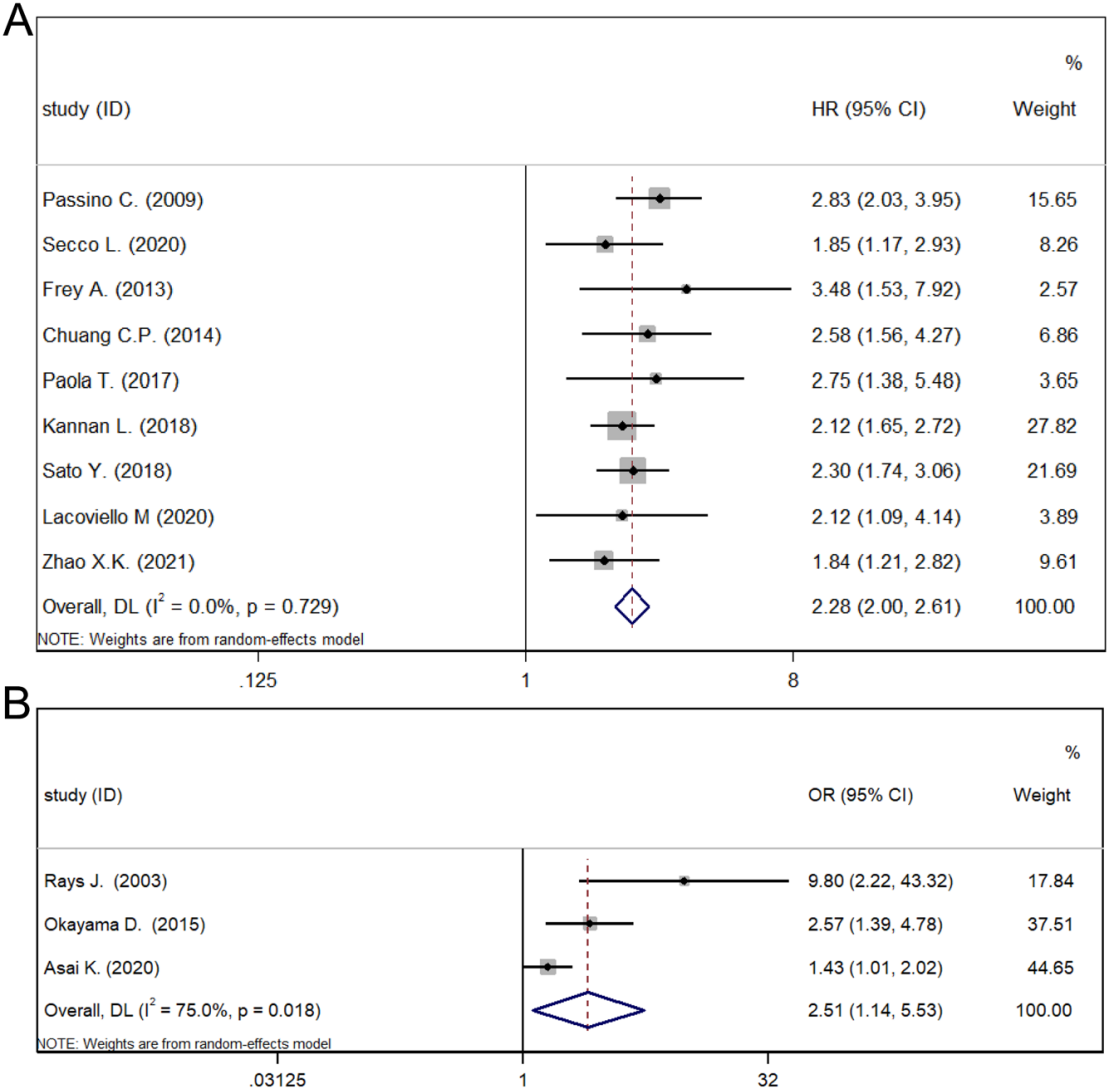
Meta-analysis of the association between NTIS and all-cause mortality of HF. (A) Meta-analysis of HRs. (B) Meta-analysis of ORs.

Of the 12 studies that explored the association between NTIS and all-cause mortality in HF patients (Fig. 2), HRs were used as the effect size in nine, while ORs were used in three. In the synthesis result of HRs, NTIS was significantly associated with an increased risk of all-cause mortality in patients with HF (HR: 2.28, 95% CI: 2.00–1.61, *P* < 0.001, *I*^2^ = 0). The pooled ORs also demonstrated a consistent result with high heterogeneity (OR: 2.51, 95% CI: 1.14–5.53, *P* < 0.001, *I*^2^ = 75%).

The results of the subgroup analyses of pooled HRs are shown in Table 2. Due to the limited number of studies, there was no subgroup analysis of ORs. For the subgroup analysis of disease onset, the pooled HRs indicated that NTIS was significantly associated with a higher all-cause mortality risk in patients with chronic heart failure (CHF) and acute heart failure (AHF) (CHF: HR: 2.32, 95% CI: 2.01–2.68, *P* < 0.001, *I*^2^ = 0; AHF: HR: 2.12, 95% CI: 1.53–2.94, *P* < 0.001, *I*^2^ = 0). For subgroup analysis based on the definition of NTIS, the synthesis of HRs showed that the association between NTIS and all-cause mortality of patients with HF was significant in both groups (strict: HR: 2.25, 95 % CI: 1.81–2.78, *P* < 0.001, *I*^2^ = 0; no strict: HR: 2.31, 95% CI: 1.95–2.73, *P* < 0.001, *I*^2^ = 0). Subgroup analysis based on the different continents of origin showed that NTIS was significantly associated with an elevated risk of all-cause mortality in studies from Europe, America, and Asia (Eur: HR: 2.50, 95% CI: 1.99–3.13, *P* < 0.001, *I*^2^ = 0; Ame: HR: 2.12, 95% CI: 1.65–2.72, *P* < 0.001, *I*^2^ = 0; Asia: HR: 2.22, 95% CI: 1.80–2.75, *P* < 0.001, *I*^2^ = 0). Finally, subgroup analysis of the source of the HRs indicated that the association between NTIS and all-cause mortality in HF patients was significant regardless of the HRs origins, that is, reported or extracted from survival curves (reported: HR: 2.24, 95% CI: 1.92–2.60, *P* < 0.001, *I*^2^ = 0; survival curves: HR: 2.36, 95% CI: 1.56–3.56, *P* < 0.001, *I*^2^ = 53.6%). However, the heterogeneity of pooled HRs from the survival curve (SC) group increased (*I*^2^ = 53.6%).

**Table 2:** Subgroup analysis of all-cause mortality.

Subgroup	No. of studies	HR (95%CI)	*P*-value	Heterogeneity		
				*I*^2^	*P*-value	Model
Disease onset						
AHF	2	2.12 (1.53–2.94)	<0.001	0	0.319	Random
CHF	7	2.32 (2.01–2.68)	<0.001	0	0.672	Random
Definition						
Strict	4	2.25 (1.81–2.78)	<0.001	0	0.647	Random
No-strict	5	2.31 (1.95–2.73)	<0.001	0	0.466	Random
Continents						
European	5	2.50 (1.99–3.13)	<0.001	0	0.539	Random
America	1	2.12 (1.65–2.72)	<0.001	0	/	Random
Asia	3	2.22 (1.80–2.75)	<0.001	0	0.567	Random
Source of HR						
Reported	7	2.24 (1.92–2.60)	<0.001	0	0.834	Random
SC	2	2.36 (1.56–3.56)	<0.001	53.60%	0.142	Random

AHF, acute heart failure; CHF chronic heart failure; HR, hazard ratio; SC, survival curves.

A sensitivity analysis was performed to assess the stability of the pooled HRs/ORs by omitting each study. The association between NTIS and all-cause mortality of HF patients remained significant after the removal of each study (*P* < 0.05) (Supplementary Fig. 1, see section on supplementary materials given at the end of this article). Funnel plots and Egger’s test indicated no publication bias for pooled HRs and ORs (*P* > 0.05) (Supplementary Fig. 3).

### Association between NTIS and MACE

**Figure 3:**
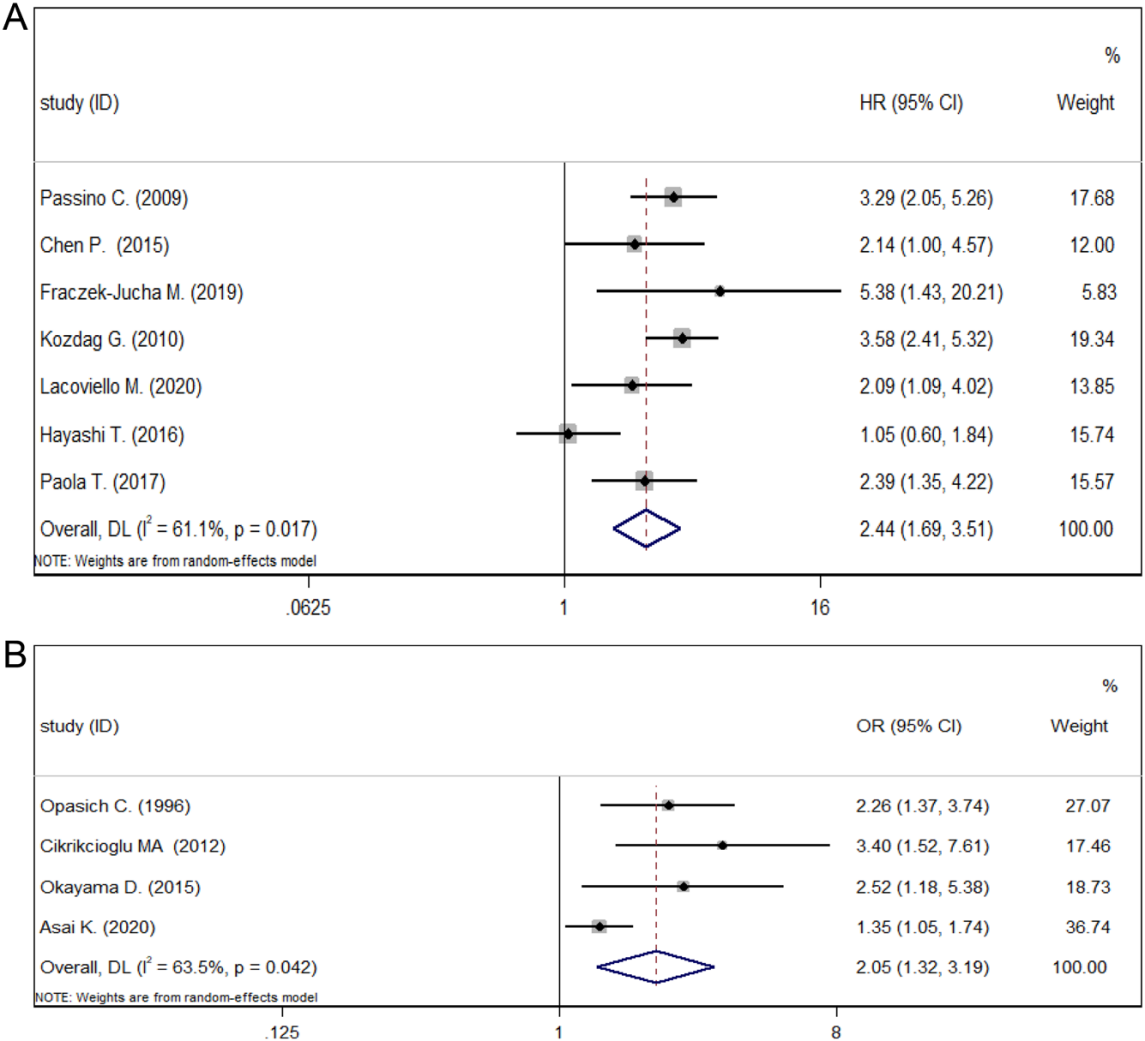
Meta-analysis of the association between NTIS and MACE in HF. (A) Meta-analysis of HRs. (B) Meta-analysis of ORs.

Eleven studies discussed the association between NTIS and MACE in HF patients (Fig. 3). The synthesis of HRs from seven studies showed that NTIS was significantly associated with a higher risk of MACE in patients with HF (HR: 2.44, 95% CI: 1.69–3.51, *P* < 0.001, *I*^2^ = 61.1%). Further, pooled ORs also confirmed a significant association between NTIS and MACE in HF patients (OR: 2.05, 95% CI: 1.32–3.19, *P* < 0.05, *I*^2^ = 63.5%). The heterogeneity of the pooled HRs and ORs was significant (*I*^2^ > 50%), so we conducted the following subgroup analyses (Table 3).

**Table 3:** Subgroup analysis of MACE.

Subgroup	No. of studies	HR (95%CI)	*P*-value	Heterogeneity		
				*I*^2^	*P*-value	Model
Disease onset						
AHF	1	1.05 (0.60–1.84)	0.500	0	/	Random
CHF	6	2.96 (2.35–3.73)	<0.001	0	0.523	Random
Definition						
Strict	3	1.72 (1.02–2.89)	0.041	56.50%	0.100	Random
No-strict	4	3.31 (2.51–4.36)	<0.001	0	0.586	Random
Continents						
European	4	2.78 (2.04–3.78)	<0.001	0	0.486	Random
America	1	3.58 (2.41–5.32)	<0.001	0	/	Random
Asia	2	1.43 (0.72–2.85)	0.312	54.20%	0.139	Random
Source of HR						
Reported	3	1.72 (1.02–2.89)	0.041	56.50%	0.100	Random
SC	4	3.31 (2.51–4.36)	<0.001	0	0.586	Random
Type of MACE						
MAE	2	3.70 (2.53–5.41)	<0.001	0	0.563	Random
Readmission for HF	2	2.11 (1.29–3.47)	0.003	0	0.963	Random
Cardiac death + readmission	2	1.58 (0.71–3.54)	0.265	75.50%	0.043	Random
Cardiac death	1	3.29 (2.05–5.26)	<0.001	0	/	Random

AHF, acute heart failure; CHF, chronic heart failure; HR, hazard ratio; MAE, major adverse events (including cardiac arrest, cardiac death, readmission due to heart failure, ventricular tachycardia, severe bradycardia); SC, survival curves.

The subgroup analysis of disease onset showed that NTIS was significantly associated with a higher risk of MACE in CHF patients (HR: 2.96, 95% CI: 2.35–3.73, *P* < 0.001, *I*^2^ = 0) but not in AHF patients (HR: 1.05, 95% CI: 0.60–1.84, *P* < 0.500, *I*^2^ = 0). For subgroup analysis based on the definition of NTIS, the association between NTIS and MACE in HF patients was significant under both strict and no-strict definition (strict: HR: 1.72, 95% CI: 1.02–2.89, *P* = 0.041, *I*^2^ = 56.5%; no-strict: HR: 3.31, 95% CI: 2.51–4.36, *P* < 0.001, *I*^2^ = 0). Subgroup analysis based on the continents of origin demonstrated that NTIS was significantly associated with an elevated risk of MACE in HF patients in studies from Europe and America (Eur: HR: 2.78, 95% CI: 2.04–3.78, *P* < 0.001, *I*^2^ = 0; Ame: HR: 3.58, 95%CI 2.41–5.32, *P* < 0.001, *I*^2^ = 0), but not significant in studies from Asia (HR: 1.43, 95% CI: 0.72 -2.85, *P* = 0.312, *I*^2^ = 54.2%). Further subgroup analysis on the source of HR indicated the significant association between NTIS and MACE in HF patients with both reported and extracted HRs (reported: HR: 1.72, 95% CI: 1.02–2.89, *P* = 0.041, *I*^2^ = 56.5%; SC: HR = 3.31, 95% CI: 2.51–4.36, *P* < 0.001, *I*^2^ = 0). For the subgroup analysis of the type of MACE, the result showed that NTIS was significantly associated with an increased risk of MACE for major adverse events (MAE), readmission for HF, and cardiac death (MAE: HR: 3.70, 95% CI: 2.53–5.41, *P* < 0.001, *I*^2^ = 0; readmission: HR: 2.11, 95% CI: 1.29–3.47, *P* = 0.003, *I*^2^ = 0%; cardiac death: HR: 3.29, 95% CI: 2.05–5.26, *P* < 0.001, *I*^2^ = 0%). However, the association was not significant in the subgroup of cardiac death and readmission for HF (HR: 1.58, 95% CI: 0.71–3.54, *P* = 0.265, *I*^2^ = 75.5%). Additionally, the heterogeneity of subgroups on disease onset, definition of NTIS, continents, and source of HR decreased compared to the pooled result, which suggests the potential source of heterogeneity.

Sensitivity analysis confirmed the stability of pooled HRs and ORs (Supplementary Fig. 2). The funnel plot and Egger’s test did not reveal a tendency for publication bias (*P* > 0.05) (Supplementary Fig. 4).

### Association between NTIS and in-hospital mortality

**Figure 4:**
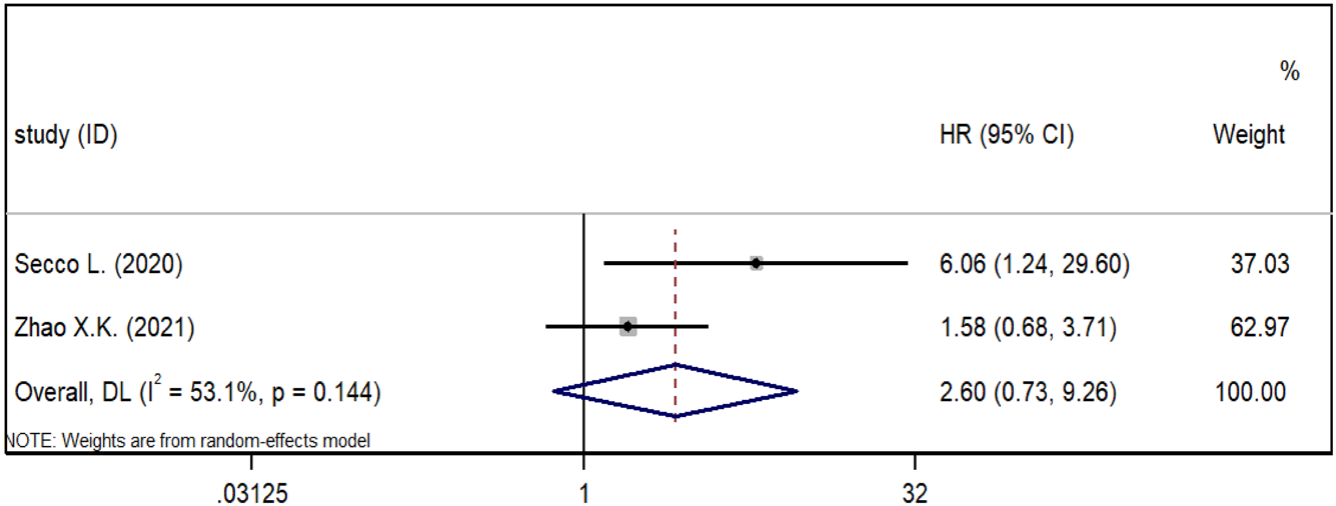
Meta-analysis of the association between NTIS and in-hospital mortality in HF.

Two studies that reported endpoints of in-hospital mortality in HF patients were included in the meta-analysis (Fig. 4). The pooled HRs showed the association between NTIS and in-hospital mortality of HF patients was not significant (*P* > 0.05). We did not perform subgroup and sensitivity analysis due to the limited number of studies. The Egger’s test revealed a potential publication bias (*P* < 0.05), but the use of the trim and fill method did not produce a reversion of the pooled result (*P* = 0.03, random-effect model).

## Discussion

In this meta-analysis, we explored the association between NTIS and both short- and long-term outcomes of HF patients. The results indicate that NTIS is significantly associated with an increased risk of all-cause mortality, and MACE in HF patients, whereas the association with in-hospital mortality was not significant. In addition, the association between NTIS and all-cause mortality remained significant in several subgroups. However, the association between NTIS and MACE was not significant in the subgroup of AHF patients, studies from Asia and MACE defined as cardiac death and readmission to hospital.

In contrast to the earlier meta-analysis focusing on the association between NTIS and cardiovascular disease, we discussed NTIS and HF prognosis. Additionally, we analyzed the association between NTIS and multiple outcomes of HF with more studies included. In the original studies, the effect size reporting the association between NTIS and HF prognosis was not uniform. To avoid potential bias, we performed separate meta-analyses for HRs and ORs. The pooled HRs and ORs confirmed that the association between NTIS and all-cause mortality, and MACE in HF patients was significant. There was no heterogeneity in the pooled HRs for all-cause mortality. In the subgroup analysis based on the source of HRs, the heterogeneity increased in the pooled HRs extracted from survival curves, which might be due to the differences in the ethnic and exclusion criteria of the two studies. However, after the exclusion of HRs extracted from survival curves, the result still supports the association of NTIS with HF prognosis. On the other hand, the meta-analysis of MACE revealed significant heterogeneity. However, the heterogeneity among subgroups including disease onset, definition of NTIS, source of HR, and continents of origin, was not significant, which indicated the heterogeneity source. Additionally, the association between NTIS and MACE in HF patients was not significant in several subgroups. The association was not significant in AHF patients, which may be explained by the limited number of studies, or a difference in the disease prognosis profile. A survey by the European Society of Cardiology (ESC), the ESC-HF pilot study survey, revealed that, compared with AHF, CHF had a better medium-term prognosis due to more mature therapies (12). Further studies will be needed to clarify this. Overall, this study was a comprehensive meta-analysis which confirmed the association between NTIS and HF prognosis. The results of the sensitivity analysis and lack of publication bias support the robustness of our conclusions.

Thyroid hormone (TH) plays a central role in regulating the cardiovascular system. Thyroxine is converted to the biologically active form of TH, triiodothyronine (T_3_), by the deiodinase enzyme (13). By binding to the nuclear receptor, T_3_ upregulates the expression of genes encoding for sodium/potassium-transporting ATPase, myosin heavy chain (MHC) α, and calcium ATPase protein of the sarcoplasmic reticulum (SERCA2α), which enhance systolic and diastolic cardiac function. Indirect effects of T_3_ on the cardiovascular system include the activation of sodium, potassium, and calcium membrane channels, which induces the endothelial nitric oxide production, and the activation of PI3K/AKT pathways to reduce vascular resistance (13). Past studies considered NTIS an adaptive mechanism to reduce metabolic demand during HF (14). Under the pathophysiologic condition of HF, increased type 3 deiodinase activity could account for the low T_3_ state (15). The persistent alterations in TH metabolism could promote HF progression through modifications in cardiac histology, morphology, and gene expression (16). A study using a rat HF model revealed that HF rats with NTIS exhibited β-MHC expression but not α-MHC expression (17). Except for the expression change of the myosin chain, the SERCA2/phospholamban ratio also decreased in HF models (16). These alterations of cardiac genes are termed ‘fetal recapitulation’ and further accelerate HF progression. Physiological T_3_ concentrations inhibit the process of fetal recapitulation, while low T_3_ levels do not (18). Furthermore, the TRα1 (thyroid hormone receptor) isoform is thought to be a molecular switch, assisting the cardiac remodeling process in HF (18). Therefore, more recent studies suggest NTIS is a maladaptive mechanism in HF, and the bidirectional association between NTIS and HF is considered a vicious cycle (14). Our meta-analysis provides clinical evidence that NTIS is associated with HF prognosis. Further basic and clinical research is necessary to determine whether it could be a therapeutic target for HF.

Our meta-analysis had several limitations. First, there were different effect sizes reported by the included studies, which limited the number of available for synthesizing pooled ORs. Further, some HRs were extracted from survival curves. Therefore, we performed separate meta-analyses for HRs and ORs and a subgroup analysis based on the source of HRs. Second, due to a lack of clinical information, we could not further analyze the association between NTIS and the prognosis of different subtypes of HF, such as HF with reduced ejection fraction and HF with preserved ejection fraction. These deficiencies require more clinical evidence to fill the gaps.

## Conclusions

In conclusion, our meta-analysis demonstrated that NTIS was significantly associated with all-cause mortality and MACE in HF patients. There was no significant association between NTIS and in-hospital mortality of HF patients. These findings suggest NTIS could be used in risk stratification for HF prognosis.

## Supplementary Materials

Supplementary Material

Supplementary Data

## Declaration of interest

The authors have no conflicts of interest to declare that are relevant to the content of this study.

## Funding

This work was sponsored by Shenzhen Science and Technology Innovation Foundation (No. JCYJ20180228162359914) and Guangdong-Shenzhen Joint Fund Youth Project (No. 2021A1515111110).
