# Supplementary material for: Non-thyroidal illness syndrome and the prognosis of heart failure: a systematic review and meta-analysis
Source: Endocr Connect. 2023 Jul 5;12(8):e230048. doi: 10.1530/EC-23-0048 (PMC10388663; doi:10.1530/EC-23-0048)
Supplement: Supplementary Material [file supplementary_material.pdf]

Supplementary table 1. Further information of included studies.

Study	Disease onset	Definition of NTIS	Exclusion criteria	Adjusted covariates
Opasich C. 1996	CHF	TT3 lower than the lowest normal limit with a normal TSH	Previous thyroid disorder; thyroid replacement therapy; amiodarone use	No adjusted
Rays J. 2003	CHF	TT3 lower than the lowest limit	Previous thyroid disorder; thyroid replacement therapy; amiodarone use	Adjusted for age, gender, NYHA class, LVEF, BMI, hypertension, diabetes, sodium, creatinine, FT4
Passino C.2009	CHF	FT3 lower than the lowest limit	Acute coronary syndrome; drug history	No adjusted
Kozdag G.2010	CHF	FT3 lower than the lowest limit	Severe systemic disease; thyroid disorders; drug history	No adjusted
Cikrikcioglu MA. 2012	AHF	FT3 lower than the lowest limit	Acute coronary syndrome; tumor; previous endocrine disease	Adjusted for BMI, EF, serum albumin, BNP, D-dimer, eGFR, absolute white blood cell counts
Frey A.2013	CHF	FT3 lower than the lowest limit; TSH and FT4 within the normal range	Incomplete results of thyroid test; drug history	Adjusted for age
Chuang C.P.2014	AHF	TT3 lower than the lowest limit	Latent thyroid disorder; Renal failure; amiodarone therapy; severe systemic disease	No adjusted

Chen P.2015	CHF	FT3 lower than the lowest limit	Past thyroid disorder; amiodarone use; acute coronary syndrome; systemic disease	No adjusted
Okayama D.2015	AHF	FT3 lower than the lowest limit	Acute coronary syndrome; aged less than 20; missing thyroid hormone measurements; thyroid disease	Adjusted for age, sex, BMI, blood pressure, BNP, sodium, creatinine, BUN, CRP, hemoglobin
Hayashi T.2016	AHF	FT3 lower than the lowest limit and TSH within the normal range	Not thyroid function test; thyroid medication; amiodarone use	Adjusted for age, heart rate, eGFR, hemoglobin
Paola T.2017	CHF	FT3 lower than the lowest limit without changes in TSH	Previous thyroid disease	/
Kannan L.2018	CHF	TT3 below the reference range and TSH/FT4 within the reference range	Taking medications would interfere with thyroid function; no informed consent	Adjusted for age, gender, race, BMI, heart failure cause, amiodarone use
Sato Y.2018	CHF	FT3 lower than the lowest limit	Amiodarone use; thyroid hormones use; anti-thyroid drugs; thyroid operation or rediation	No adjusted
Fraczek-Jucha M.2019	CHF	FT3 below the normal range	Systemic disease; thyroid dysfunction; treatment with amiodarone	No adjusted
Secco L.2020	CHF	TT3 lower than the lowest limit	Thyroid disease; acute coronary syndrome; thyroid hormone replacement; severe systemic disease	No adjusted

Asai K.2020	AHF	FT3 lower than the lowest limit	HF caused by acute coronary syndrome; lack of thyroid function data	Adjusted for age, gender, blood pressure, LVEF, BNP, hemoglobin, bilirubin, sodium
Lacoviello M.2020	CHF	TT3 lowers the lowest limit and TSH is within the normal range	Not mentioned	Adjusted for age, etiology, diabetes, therapy, LVEF, BNP
Zhao X.K.2021	AHF	FT3 lower than the lowest limit	Thyroid disorder; use of anti-thyroid drugs/thyroid hormone/amiodarone; acute coronary syndrome	Adjusted for blood urea nitrogen, blood pressure, BMI, NYHA class, sodium, albumin

AHF: acute heart failure; CHF: chronic heart failure; TT3: total triiodothyronine; FT3: free triiodothyronine; FT4: free thyroxine; TSH: thyroid-stimulating hormone; MACE: major cardiovascular events; BMI: body mass index; NYHA: NewYork Heart Association; BNP: B-type natriuretic peptide; BUN: blood urea nitrogen; CRP: C-reactive protein; eGFR: estimated glomerular filtration rate; LVEF: left ventricular ejection fraction

Supplementary figure 1. Sensitivity analysis of all-cause mortality. (A: sensitivity analysis of pooled ORs;

B: sensitivity analysis of pooled HRs)

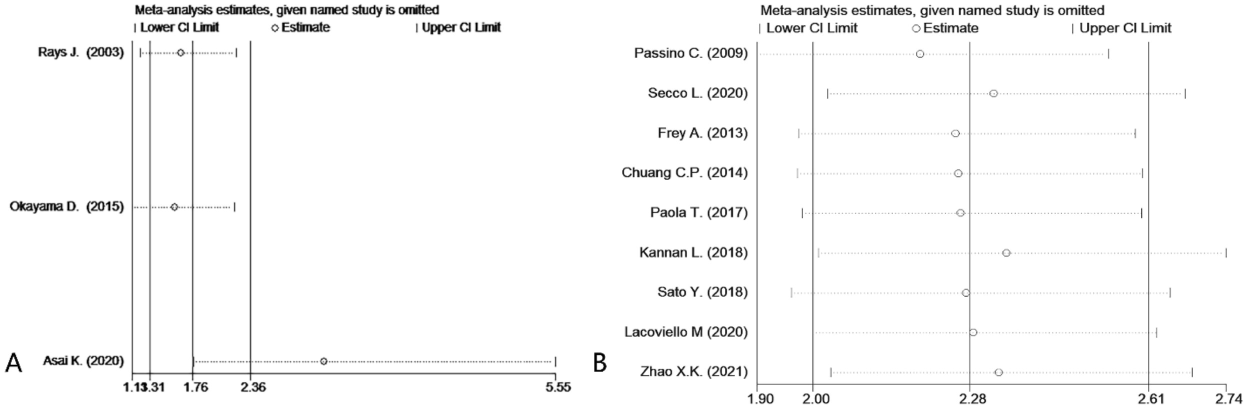

Supplementary figure 2. Sensitivity analysis of MACE. (A: sensitivity analysis of pooled ORs; B: sensitivity analysis of pooled HRs)

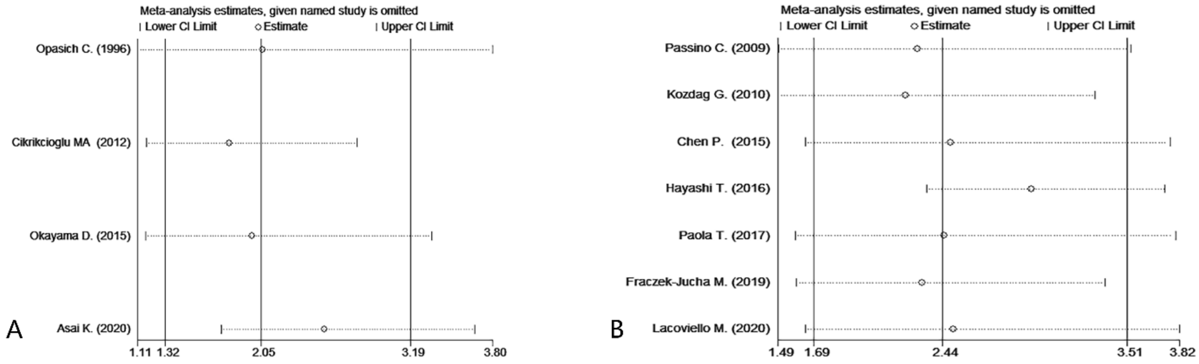

Supplementary figure 3. Funnel plot of all-cause mortality (A: funnl plot of HRs; B: funnel plot of ORs)

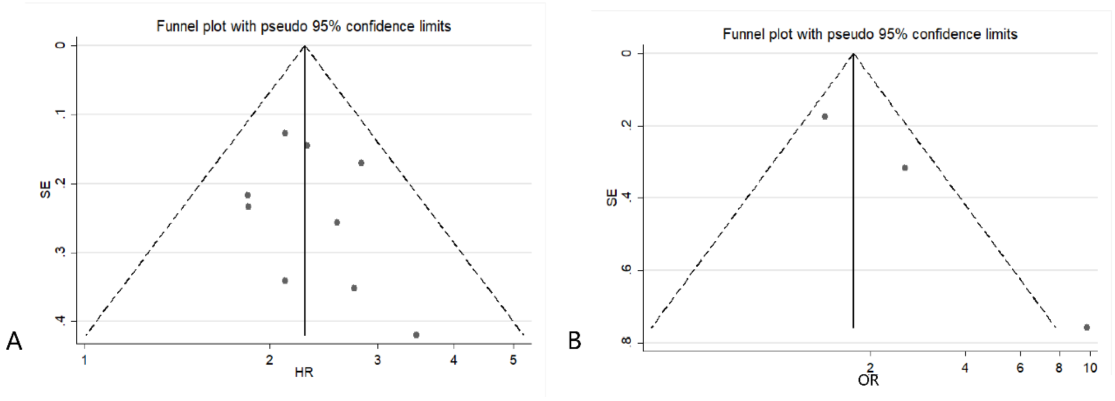

Supplementary figure 4. Funnel plot of MACE (A: funnl plot of HRs; B: funnel plot of ORs)

A

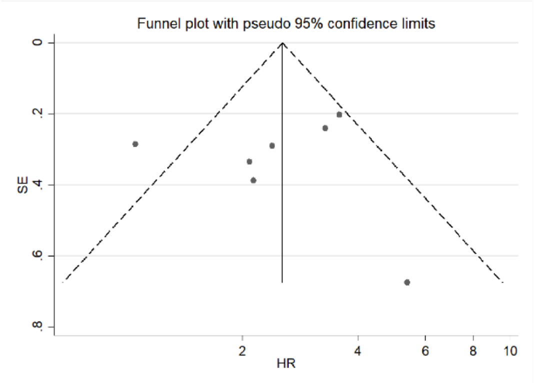

B

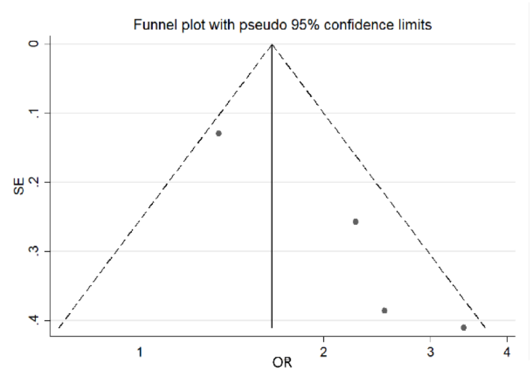

OR
